# Heterogeneity within phycobilisomes is highly orchestrated

**DOI:** 10.1007/s11120-026-01202-8

**Published:** 2026-02-09

**Authors:** Jaspreet K. Sound, Maayan Suissa Szlejf, Hannah E. Wedgwood, Noam Adir, Aneika C. Leney

**Affiliations:** 1 School of Biosciences, University of Birmingham, Edgbaston, Birmingham, B15 2TT UK; 2 Schulich Faculty of Chemistry, Technion, Haifa 3200003 Israel

**Keywords:** Cyanobacteria, Light-harvesting complexes, Phycobilisome, Structural heterogeneity, Native mass spectrometry

## Abstract

The phycobilisome (PBS) is one of the oldest and most efficient light-harvesting protein complexes known. Throughout billions of years of evolution, the PBS has readily adapted to its environment through differential gene expression alongside controlled assembly/disassembly of its components. How hundreds of protein subunits assemble in a controlled manner into different PBS structures within different cyanobacterial species is unknown. Moreover, PBSs are not static entities, and can have different compositions that enables them to modulate their overall function in response to changes in their environment. Here, we utilised high resolving native mass spectrometry (MS) to probe the heterogeneity within PBSs and determine factors that govern its self-assembly. By monitoring PBSs stable sub-complexes, we were able to detect low abundant allophycocyanin variants ApcD and ApcF, in addition to the core linker protein ApcC, and determine their complex stoichiometry. Furthermore, native MS revealed the phycobiliproteins, phycocyanin and allophycocyanin, are unable to form mixed rings within the PBS structure, and instead can only stack, one on of top of another, for efficient energy transfer. Finally, we show that in strains such as *A. marina* where no allophycocyanin core is present, phycocyanin variants acts as distinct entities to ensure highly orchestrated PBS assembly. Together, the methodology and data obtained open up new avenues for future exploration into how the PBS adapts for effective function.

**Supplementary Information:**

The online version contains supplementary material available at 10.1007/s11120-026-01202-8.

## Introduction

Photosynthetic organisms are terrific in their ability to capture and transfer solar energy to dedicated chlorophyll-containing photochemical reaction centres for conversion to electron and proton transfer reactions required for all of the cell’s metabolic needs. Cyanobacteria and red microalgae, in particular, possess specialised light harvesting complexes, termed phycobilisomes (PBS), which extend the range of solar radiation absorbed from 450 to 670 nm which enhances their photosynthetic efficiency (Bryant and Gisriel 2024; Adir et al. 2020; Zhao et al. 2025). This broad spectrum of light energy absorbed by the PBS, is then transmitted to the photosystem II reaction centre (or photosystem I under specific acclimation states), whereby its light energy is rapidly converted to chemical energy. Due to the evidence of the survival of cyanobacteria across billions of years of evolution (Sánchez-Baracaldo et al. 2022), it appears that PBS are highly adaptable and can function effectively in a variety of environments, such as freshwater, saltwater, in soil, as well as at a range of temperatures (Makhalanyane et al. 2015; Oren 2015).

**Fig. 1:**
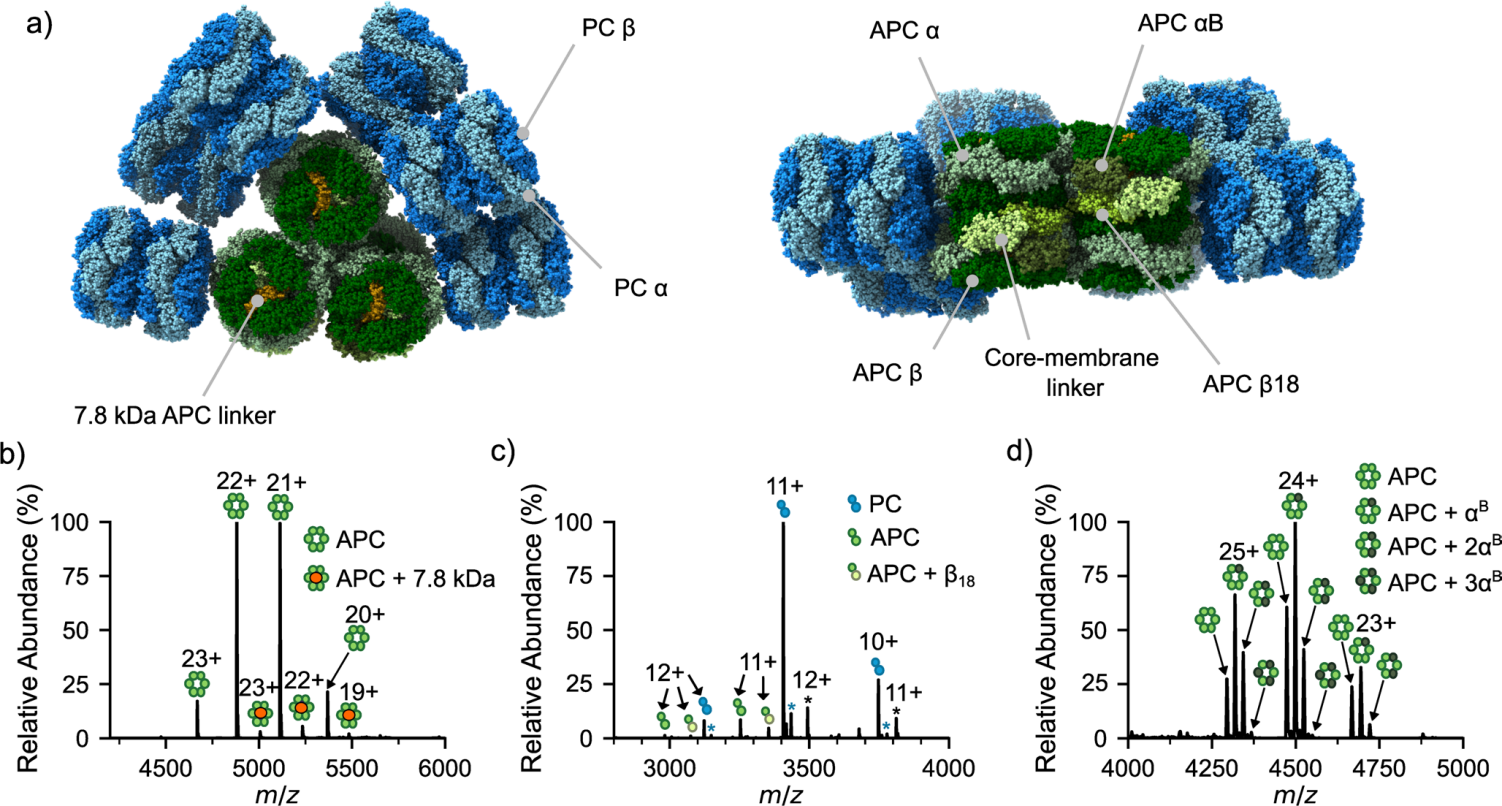
(**a**) top (left) and bottom view (right) of the tricylindrical hemidiscoidal PBS from *Synechococcus* sp. PCC 7002 (PDB: 7EXT) showing location of the phycocyanin rods (PC, blue) and allophycocyanin core (APC, green) including its components β_18_ (ApcF), α^B^ (ApcD), the core-membrane linker (ApcE) and the 7.8 kDa linker ApcC protein (orange). Native MS of (**b**) (αβ)_3_ APC in association with the 7.8 kDa APC linker protein (ApcC), (**c**) the αβ_18_ hetero-dimeric APC (ApcA-ApcF) complex, and (**d**) (αβ)_3_ APC complexes containing up to 3 copies of the α^B^ variant (ApcD). Peaks in (**c**) marked with an asterisk correspond to PC αβ dimer with modification (blue asterisk) and phosphoglycerate kinase (black asterisk), respectively

Phycobilisomes range from 1 to 18 MDa in size (Hu et al. 1999; Zhang et al. 2017), and are comprised of up to hundreds of chromophorylated proteins, termed phycobiliproteins (PBPs) (Sui 2021). In cyanobacteria, the dominant phycobiliproteins are phycocyanin (PC) and allophycocyanin (APC). APC and PC are composed of heterodimers (typically called monomers) of α and β subunits that self-assemble into stable ring-shaped (αβ)_3_ complexes. In most cases, PBS are hemi-discoidal structures where stacks of (αβ)_3_ PC (λ_max_ = 620 nm) form rod-like structures which protrude from the PBS core which is composed of cylinders of stacked (αβ)_3_ APC (λ_max_ = 652 nm), ensuring unidirectional energy transmission from the rods to the core and eventually to chlorophyll *a* (Fig. 1a). Increasing evidence suggests that within the PBS’s hemi-discoidal structure, its protein composition can vary. These properties have ensured optimal photosynthetic function across large evolutionary timescales, yet how this heterogeneity is controlled is still poorly understood.

Heterogeneity within PBSs is particularly evident within the APC core wherein APC subunit variants substitute in and out of the PBS to enable it to adapt rapidly to variable light intensities, as well as different wavelengths of light (Gan and Bryant 2015; Soulier et al. 2020, 2022; Ho et al. 2020). The main APC variants consist of α^B^ (ApcD) and β_18_ (ApcF), with ApcG more recently identified. α^B^ has been shown to swap with a single APC α subunit within certain (αβ)_3_ complexes, whilst β_18_ replaces a single APC β subunit and interacts with the core-membrane linker protein, ApcE (which also replaces a single APC α subunit, Fig. 1a). Complexes containing α^B^ and/or β_18_ are typically found in lower copy numbers within the PBS, yet aid significantly in extending the emission wavelength to ~ 670–680 nm (Gindt et al. 1994; Glazer and Bryant 1975; Chen et al. 2025; Gisriel et al. 2024) which facilitates the terminal emission of energy from the PBS to photosystem I/II (Ashby and Mullineaux 1999; Calzadilla et al. 2019). In addition to PBPs, linker proteins are found throughout the entire PBS, adjoining different PBP complexes. Two linker proteins, the 7.8 kDa protein ApcC and ~ 100 kDa linker ApcE, are found within the hemi-discoidal PBS core wherein ApcE, both contributes to the terminal emission of energy to PS I/II and tethers the PBS to the thylakoid membrane (Liu et al. 2021). However, not all cyanobacteria contain hemi-discoidal PBSs. Moreover, alternative arrangements such as single rod, rod-bundle and paddle-shaped PBS (Bryant and Gisriel 2024; Jiang et al. 2023; Guglielmi et al. 1981; Burtseva et al. 2025) also exist likely containing their own balance of heterogeneous sub-complexes. For example, the single rod PBS of the cyanobacterium *Acaryochloris marina MBIC11017* (*A. marina*) consists of single PC rods (Hu et al. 1999; Marquardt et al. 1997; Suissa Szlejf 2025). Yet, despite its smaller size, *A. marina* PBS are unique in that they co-express four genes that result in the presence of two isoforms for both the α and β subunits (Bar-Zvi et al. 2018). *A. marina* also uniquely produces chlorophyl *d* that acts to aid the absorption of far-red light (Miyashita et al. 1997). Deciphering to what extent PBSs use PBPs heterogeneity to control and adapt their function is only beginning to be understood. Moreover, structural biology techniques, such as cryo-electron microscopy and X-ray crystallography frequently overlook heterogeneities due to the averaging over immense numbers of molecules.

Native mass spectrometry excels in its ability to monitor samples heterogeneity and capture transient protein complexes of low abundance (Reid et al. 2023; Rolland and Prell 2022; Veale and Clarke 2024). By effectively transferring non-covalent complexes from solution through to analysis, information can be reported on complex stoichiometry and architecture (Leney and Heck 2017; Karch et al. 2022; Tamara et al. 2022). Specific to PBSs, native MS has been used to monitor PBPs complex assembly (Leney et al. 2018; Leney 2019), phycocyanin fluorescence quenching upon metal binding (Bellamy-Carter et al. 2022), concentration-dependent PC assembly (Eisenberg et al. 2017), and also characterize Orange Carotenoid Protein and its domains (Zhang et al. 2016) that binds to the PBS in some organisms, protecting the cyanobacteria from light-induced damage. More recently, native MS has been applied to explore the evolutionary divergence of PBPs by monitoring the formation of heterologous complexes containing PBPs from diverse cyanobacterial species (Sound et al. 2026). Although native MS analysis of PBS sub-components has proven effective, its ability to probe heterogeneity within single PBS is yet to be explored. Here, we show native MS is highly efficient as probing PBS heterogeneity. We show that native MS can readily detect low abundant components within the APC PBS core. By monitoring the PBS sub-complexes that exist in vitro, we show that complex formation is highly orchestrated. Moreover, although heterogeneous complexes can readily form with variants of the APC core, when two variants of the α and β PC subunits were co-expressed within *A. marina*, no heterogeneous complexes comprising one of each α/β isoforms (i.e. α_1_β_2_ or α_2_β_1_) were observed.

## Materials and methods

### Cyanobacterial growth

*Arthrospira platensis* (*A. platensis*) was supplied from Algae Research Supply (US) and *Spirulina major* (*S. major*) was supplied from CCAP (Oban, Scotland). Both strains were grown in 50:50 ASW: BG11 medium on a rocker at room temperature under a 12:12 h light/dark regime. *Acaryochloris marina MBIC11017* (*A. marina*) were grown under constant low white light (~ 5 µmol E) in MBG11 medium supplemented with 5% CO_2_ in air at room temperature. Under these conditions, all genes encoding α_1_, β_1_, α_2_ and β_2_ are known to be expressed (Bar-Zvi et al. 2018).

### Phycobiliprotein extraction and purification

Cyanobacterial cell pellets (three unique biological samples) were lysed by the addition of ultra-pure water and several rounds of freeze-thaw (-70 to + 25 °C). The use of ultra-pure water lowered the ionic strength to promote PBS disassembly into its PBP components. Successful lysis was determined by the presence of a red-fluorescing supernatant upon excitation with a torch, indicative of phycobiliprotein presence. The cell lysates containing PBPs were then centrifuged (10,500 x*g*, 10 min, 4 °C) to remove cell debris.

To detect the APC β_18_ subunit (ApcF), the PBP extract from *A. platensis* was analysed directly by native MS. High purity APC was purchased from Sigma-Aldrich and analysed directly to detect the *A. platensis* 7.8 kDa APC core linker protein (ApcC). To obtain the mixture of α_1_β_1_ and α_2_β_2_ PC complexes from *A. marina*, the PBP from the cell extract were first purified using ammonium sulphate precipitation. Briefly, 25% ammonium sulphate in 5 mM potassium phosphate pH 7.4 was added to the phycobiliprotein extract and any unwanted precipitated proteins and pigments were removed. 60% ammonium sulphate in 5 mM potassium phosphate pH 7.4 was then added to precipitate the α_1_β_1_ and α_2_β_2_ PC complexes and the supernatant discarded. The precipitate was then resuspended in 5 mM potassium phosphate pH 7.4 and before analysis by native MS.

To isolate the APC and PC (αβ)_3_ complexes from *A. platensis* and *S. major*, cell lysis and ammonium sulphate precipitation was performed as described above. The APC and PC (αβ)_3_ complexes were then separated by anion exchange chromatography using a self-packed CHT Ceramic Hydroxyapatite column (Type 1, 40 μm) (Bio-Rad), pre-equilibrated in 5 mM potassium phosphate pH 7.4. Purification was performed on an ÄKTA Pure 25 chromatography system (Cytiva) whereby the absorbances at 280 nm, 620 nm and 650 nm were used to monitor the elution of the phycobiliproteins. (αβ)_3_ PC interacted weakly with the column and eluted immediately after the flow through. APC (αβ)_3_ complexes eluted using 50 mM potassium phosphate pH 7.4. To isolate the APC (αβ)_3_ complexes containing α^B^ subunits, the ionic strength was further increased to 400 mM potassium phosphate pH 7.4 (Fig. S1) and the eluting fractions collected for analysis. α_2_β_2_ from *A. marina* was purified in a similar manner to PC from *A. platensis* and *S. major*. α_2_β_2_ eluted immediately after α_1_β_1_ from the anion exchange column (Fig S2). PBP purity was confirmed by MS analysis (Fig. S3, S7a). All purified PBP were stored at 4 °C in the dark in their elution buffer prior to native MS analysis. To prevent problems associated with PBP instability, once samples had been purified, native MS was performed within 48 h.

### Native mass spectrometry

Prior to native MS analysis, all PBP samples were buffer exchange into 100 mM ammonium acetate pH 6.8 using Amicon Ultra 0.5 mL concentrators with a 30 kDa molecular weight cut-off filter (Merck Millipore). The PBP samples were analyzed at a concentration of 0.5–20 µM. Due to the differing Kd’s of PC and APC’s (αβ)_3_ complexes (Bellamy-Carter et al. 2022), when APC and PC complexes were mixed, the concentration ratio of APC: PC was further adjusted to ensure the (αβ)_3_ complexes were present at the same absolute intensity.

All native MS experiments were performed on an Orbitrap Ascend Structural Biology mass spectrometer (Thermo Fisher Scientific) using a nano-electrospray ionisation source fitted with gold coated borosilicate glass capillaries pulled in-house (P-1000, Sutter Instrument). The instrument was calibrated using the Thermo Scientific Pierce FlexMix Calibration Solution. Positive ionisation mode was used without with a capillary voltage of 1.2 kV, source temperature set to 250 °C and in-source fragmentation set to zero. Mass spectra were acquired on all biological replicates in intact protein mode at high pressure. Ions were detected in the Orbitrap with a mass range set to 1000–8000 *m/z*, resolution set to 15,000, automatic gain control 100%, and the maximum injection time set to 100 ms. All spectra were acquired for > 1 min and the average spectra over this timescale reported.

The native MS data was processed using XCalibur v4.2 (Thermo Fisher Scientific). Theoretical masses of the APC and PC complexes for each strain were calculated from the amino acid sequences of the APC α, α^B^, β, β_18_ and 7.8 kDa core linker and PC α and β subunits. The theoretical masses of all PBP components were adjusted to include all previously identified post-translational modifications. The APC 7.8 kDa core linker protein contains no known post-translational modifications. For the other APC complexes, the masses of the α and α^B^ subunits were modified to include the addition of 1 phycocyanobilin (PCB) chromophore (+ 586.7 Da) and the loss of the initiator methionine (− 131.2 Da) while the masses of the β and β_18_ subunits were modified to include the addition of 1 PCB chromophore and the methylation of Asn71 to N4-methylAsn71 (+ 14 Da). For the PC complexes from *A. platensis* and *S. major*, the masses of the α subunits were modified to include the addition of 1 PCB chromophore while the masses of the β subunit were modified to include the addition of 2 PCBs with methylation of Asn72 to N4-methylAsn72. For both PC isoforms from *A. marina*, the masses of the α and β subunits were adjusted for the addition of 1 and 2 PCBs, respectively, but no methylation of Asn72 was added (Suissa Szlejf 2025). The experimentally determined molecular weights were calculated manually using multiple charge states observed for each complex (Table S1) using a defined S/N threshold of 3:1. The percentage error between the theoretical and experimentally determined molecular weights were used to verify the presence of all PBP complexes, whereby percentage errors of < 0.1% confirmed the presence of the corresponding protein complex. Low abundant complexes in the mass spectrum were defined as relative intensity < 5% of the base peak.

### Bioinformatics

The PC α and β subunits from different species were aligned using the ClustalW Multiple Alignment tool on BioEdit v7.7.1 (Hall 1999). For visualising potential inhibitory interactions between the *A. marina* α_1_β_1_-α_2_β_2_ dimers, the AlphaFold predictions of the individual α and β monomers were taken from UniProt (α_1_ = A8ZMJ4, α_2_ = A8ZMJ6, β_1_ = A8ZMJ5, β_2_ = A8ZMJ7), and these structures were aligned to the high resolution PC (αβ)_3_ complex from *A. platensis* (PDB = 1HA7) (Padyana et al. 2001). All structures were visualized with ChimeraX v1.9 (Meng et al. 2023).

## Results and discussion

### Native mass spectrometry can probe heterogeneity within phycobiliprotein sub-complexes

To probe whether native MS can detect PBP complex heterogeneity within the PBS, purified APC from *A. platensis*, a tricylindrical hemidiscoidal PBS containing cyanobacterium, was purchased and analyzed by native MS (Fig. 1b). Consistent with the ability of native MS in preserving non-covalent complexes, a charge state distribution at 4,600-5,500 *m/z* was detected corresponding to an APC trimer of αβ dimers (Fig. 1b). Alongside these peaks, lower intensity peaks were also detected corresponding to the same APC (αβ)_3_ complex but with a single 7.8 kDa APC core linker protein, ApcC, associated to it. Thus, we next sought to determine whether other APC variants could also be detected by native MS. *A. platensis* was grown under standard conditions, and all PBPs extracted and crudely purified by passing the cell lysate through a 30 kDa molecular weight cut off filter and retaining the proteins and protein complexes that were > 30 kDa in size. As expected, native MS of the PBP mixture showed high intensity peaks in the 3,000–4,000 *m/z* region corresponding to the dominant PBP PC αβ complex (Fig. 1c). An APC αβ complex and an APC αβ_18_ complex were also observed confirming that our method has the sensitivity necessary to detect minor complexes within the intact phycobilisome core of *A. platensis*.

The APC α^B^ variant has been observed within PBS cryo-electron microscopy studies, whereby a single copy has been observed integrated within a (αβ)_3_ complex (α^B^α_2_β_3_); two α^B^ subunits in total within the core (Zheng et al. 2021). A complete (α^B^β)_3_ complex has been expressed and a crystal structure was solved (Peng et al. 2014), however, this same complex stoichiometry has yet to be observed in vivo. To capture any α^B^-containing complexes present, PBPs were extracted again from *A. platensis*, but this time individual complexes were purified via a combination of ammonium sulphate precipitation followed by anion exchange chromatography. Interestingly, the later eluting, low abundant, 650 nm absorbing fractions from the anion exchange column showed an abundance of APC (αβ)_3_ complexes where the α subunit had been substituted with up to 3 α^B^ subunits (Fig. 1d). This suggests that α^B^ containing complexes can dis-assemble and re-assemble wherein multiple copies of α^B^ can be incorporated if the abundance of the α^B^ subunit is on the same order to that of the α subunit. As none of the complex variants show the presence of ApcC, this result indicates that the replacement of α subunits by α^B^ subunits does not require this linkers presence. This might also indicate that ApcC associates with a mature (α^B^α_2_β_3_) hexamer, prior to association of the next hexamer in the core cylinder.

### Complex formation is highly controlled between distinct phycobiliprotein complex components

**Fig. 2:**
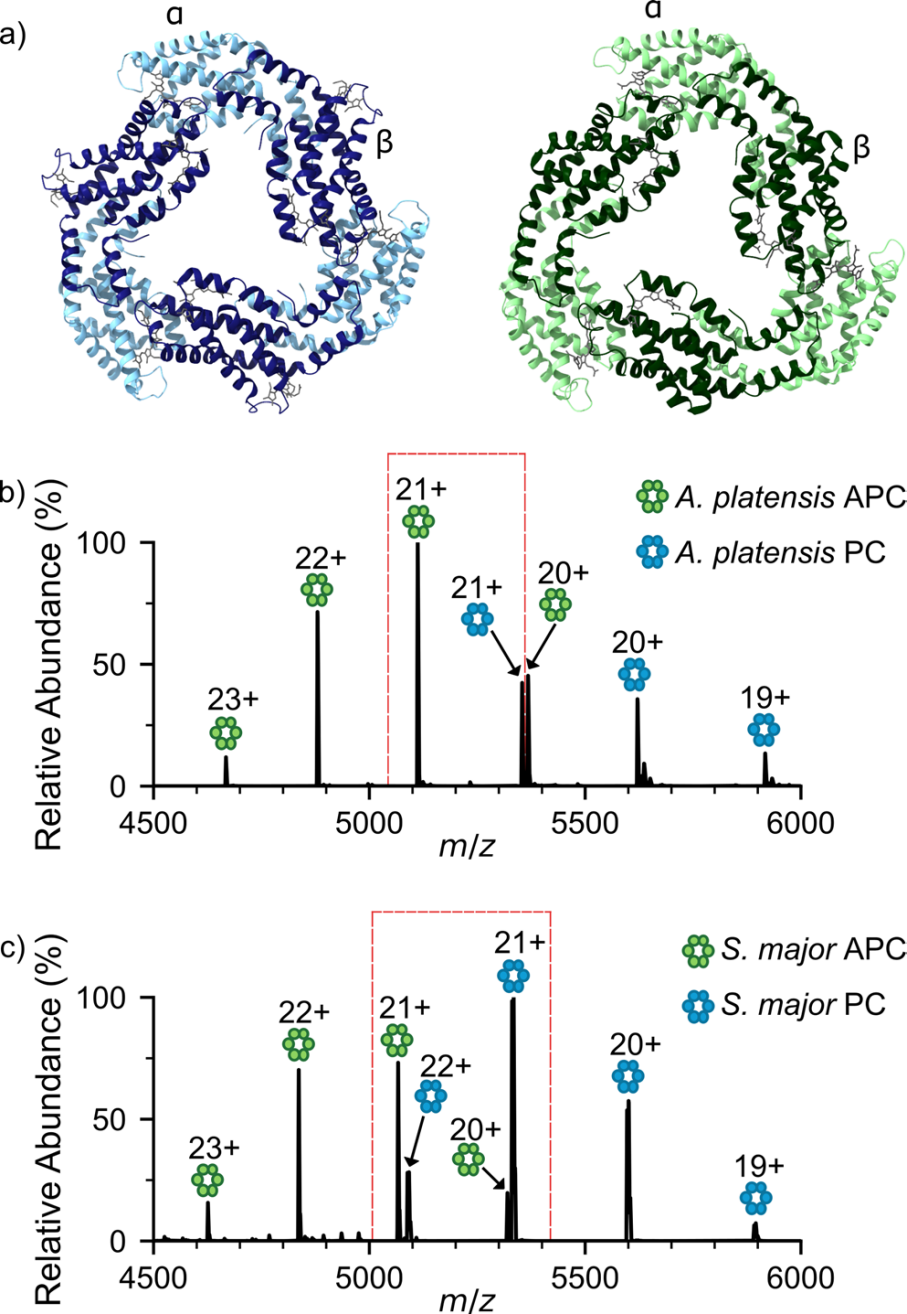
Phycocyanin and allophycocyanin are distinct units. (**a**) Structures of (αβ)_3_ PC (blue, PDB:1HA7) and (αβ)_3_ APC (green, PDB:4F0U) with PCB chromophores highlighted in grey. Native MS of mixtures of purified PC and APC from (**b**) *A. platensis* and (**c**) *S. major* show no heterogenous (αβ)_3_ complexes form. Red boxes highlight the lack of complex formation between APC and PC peaks of the same charge state

Native MS has shown that heterogeneous complexes exist within the APC core of the PBS, thus we next sought to determine whether heterogeneous complexes form comprising both the two main distinct PBP variants, APC and PC, within a single (αβ)_3_ complex. Moreover, although they have less than 40% sequence identity and distinct functions in light energy transfer, their structural similarity is remarkably conserved (Fig. 2a). PC and APC were extracted and purified from two distinct cyanobacteria species, *A. platensis* and *S. major* (Fig. S3), incubated for 1 h at a 1:1 PC: APC ratio, and the complexes formed analyzed by native MS (Fig. 2b, c). As expected, mass spectra showed clear charge state distributions corresponding to PC and APC (αβ)_3_ complexes in both species. However, no heterogeneous complexes consisting of 2αβ_APC_.αβ_PC_ (5,193 *m/z* for 21 + charge state for *A. platensis*) or 2αβ_PC_.αβ_APC_ (5,273 *m/z* for 21 + charge state for *A. platensis*) were detected (Fig. 2). Indeed, these findings are consistent with previous data whereby multiple strains of cyanobacteria were simultaneously analysed, yet no (αβ)_3_ complexes comprising both PC and APC αβ complexes were observed (Sound et al. 2021, 2026). Combined, this native MS data (in Figs. 1 and 2) highlights that the heterogeneity within the PBS is strictly regulated to ensure optimal PBS function and happens spontaneously without the need for linkers or other chaperones. Indeed, this is consistent with previous studies that have shown structural attributes that assist or prevent the formation of mixed αβ complexes (McGregor et al. 2008) and mixed (αβ)_3_ hexamers (Adir et al. 2001).

### PC isoforms within *A. marina* form structurally distinct complexes

**Fig. 3:**
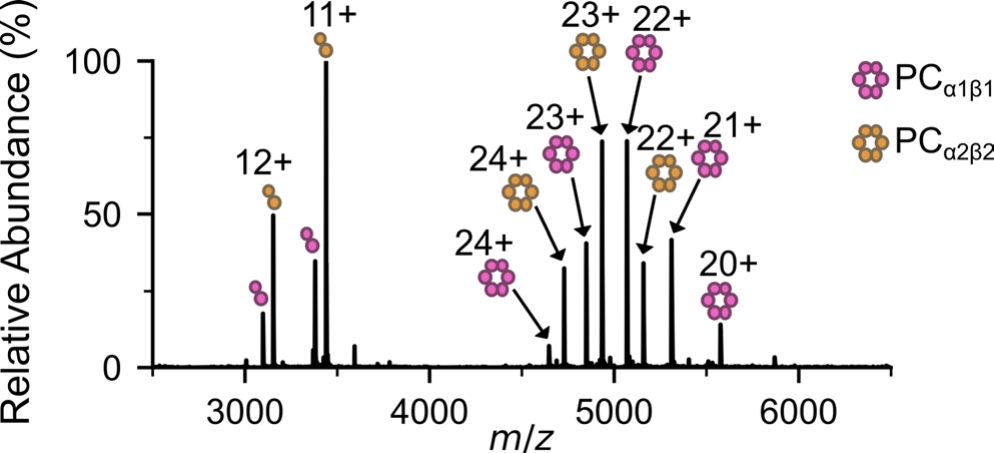
*A. marina* expresses two phycocyanin isoforms under low light conditions. Native MS of PBPs from *A. marina* shows the presence of αβ dimer and (αβ)_3_ PC complexes composed of the two different PC isoforms, α_1_β_1_ (pink) and α_2_β_2_ (orange)

**Fig. 4:**
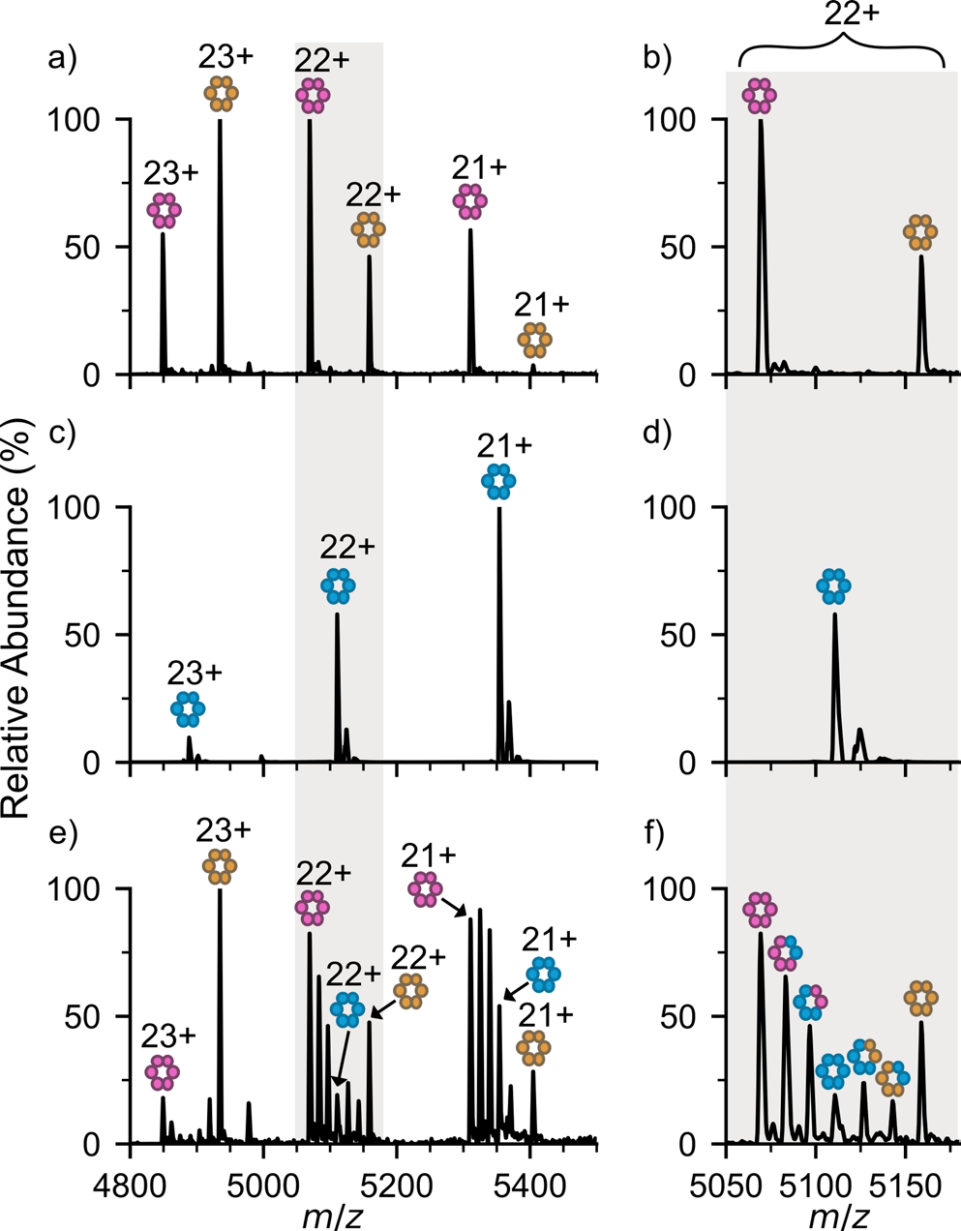
*A. marina* PC isoforms form selective heterogenous complexes. Native MS of (**a**) (α_1_β_1_)_3_ (pink) and (α_2_β_2_)_3_ (orange) PC complexes from *A. marina*, (**c**) (αβ)_3_ PC (blue) from *A. platensis* individually and (**e**) mixed together, alongside a close-up of the 22 + charge state (**b**, **d**, **f**)

*A. marina* is a unique strain of cyanobacteria in that it expresses two isoforms of both the PC α and β subunits under a variety of environmental conditions including low white light intensity. Due to their co-observed presence under denaturing, proteolytic mass spectrometry conditions, it has been hypothesised that these PC variants can interact to form multimeric (αβ)_3_ complexes comprising all four isoforms (Bar-Zvi et al. 2018; David et al. 2014). To investigate whether these indeed form, we grew *A. marina* in low light to express the two isoforms, then extracted and analysed its PBP sub-complexes by native MS (Fig. 3). Intriguingly, only α_1_β_1_ and α_2_β_2_ complexes and trimers of these complexes ((α_1_β_1_)_3_ and (α_2_β_2_)_3_) were observed (Fig. S4, S5, Fig. 4a, b). Moreover, no heterogeneous complexes (i.e. α_1_β_2_ or α_2_β_1_) were observed within the monomeric or trimeric unit, suggesting that similar to PC and APC in other strains (Fig. 2), α_1_β_1_ and α_2_β_2_ within *A. marina* also function as distinct entities. Moreover, under the conditions analysed, PC was the most abundant PBP within *A. marina*, with little to no APC observed (Fig. 3). This is consistent with previous work, wherein APC was noted to be a minor PBP component in *A. marina* (Hu et al. 1999; Marquardt et al. 1997; Bar-Zvi et al. 2018).

Previous data has shown that PC from different species can interact with one another to form heterogeneous complexes (Sound et al. 2026). Therefore, we next investigated whether α_1_β_1_ and α_2_β_2_ from *A. marina* interact with PC from other species to the same extent. *A. marina* (α_1_β_1_)_3_ and (α_2_β_2_)_3_ PC complexes were incubated with purified (αβ)_3_ PC from *A. platensis* (Fig. 4c, d) for 1 h, and the complexes formed analysed by native MS. Strikingly, although (α_1_β_1_)_3_ and (α_2_β_2_)_3_ alone do not mix, heterogeneous (αβ)_3_ complexes were observed comprising a mixture of αβ PC from *A. platensis* and either α_1_β_1_ or α_2_β_2_ from *A. marina* (Fig. 4e, f, Fig. S6). Moreover, even when highly purified α_2_β_2_ PC from *A. marina* (Fig. S7a, b) was mixed with highly purified αβ PC from *A. platensis* (Fig. S3a), heterogeneous (αβ)_3_ complexes were still detected (Fig S7c, d). This suggests that the formation of these mixed (αβ)_3_ complexes is not dependent on other PBS proteins, such as linker proteins, although these could assist in selective complex formation in vivo. Purified α_2_β_2_ PC did not form mixed complexes with purified (αβ)_3_ APC from *A. platensis* (Fig. S7e, f), highlighting that the heterogeneous interactions observed are PC-specific, and heterogeneity is not randomly observed, but instead, selectively controlled.

**Fig. 5:**
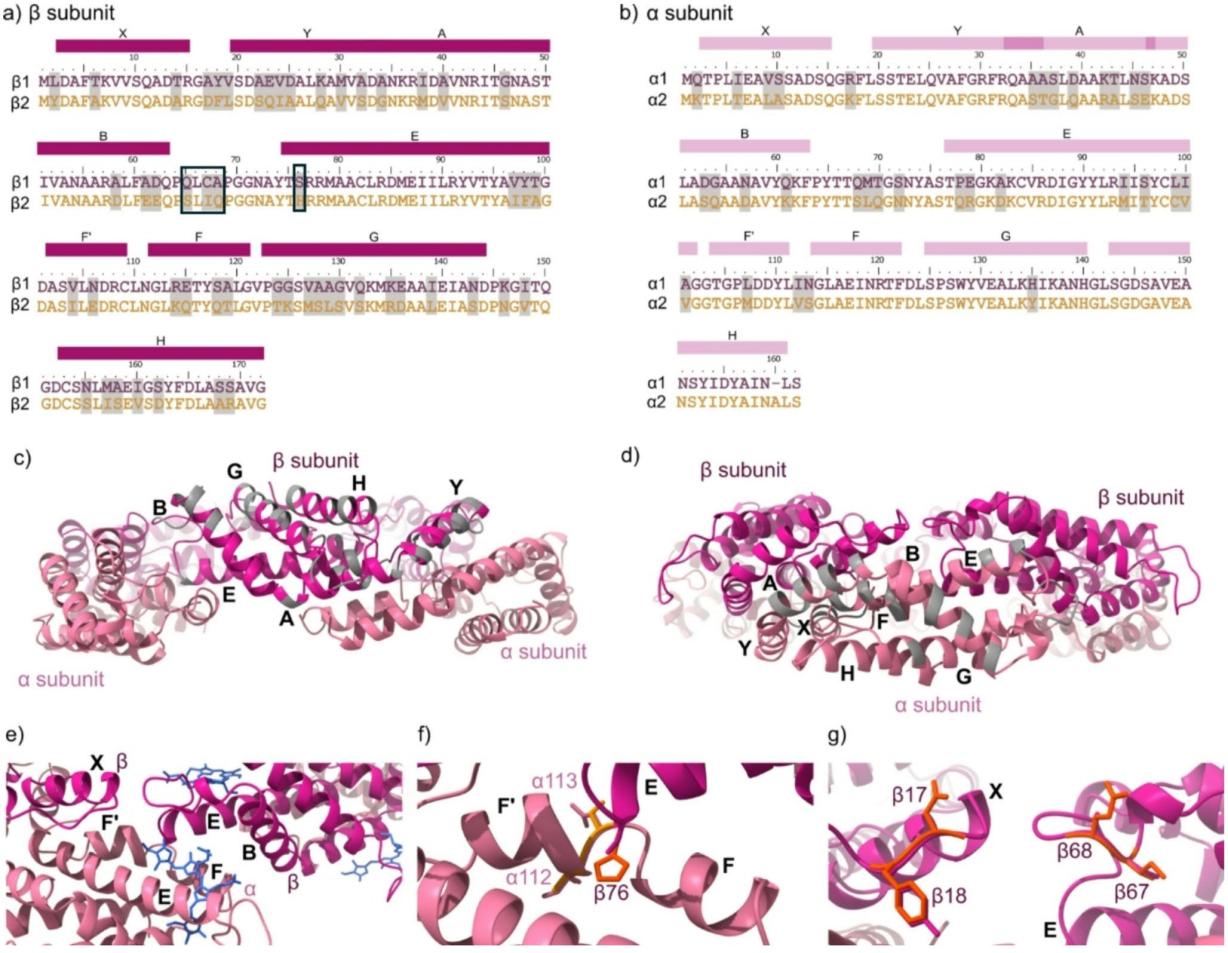
Structural alignment and predictions of PC variants. Sequence alignment of the (**a**) β subunit variants (β_1_, β_2_) and (**b**) α subunits variants (α_1_, α_2_) from *A. marina*. Regions of the sequence corresponding to the α helices are shown with coloured bars and the sequence differences between the two variants are highlighted in grey for both subunits. The AlphaFold 3 predicted structure of the *A. marina* (α_1_β_1_)_3_ hexamer shows differences between the (**c**) β_1_ and β_2_ subunits. and the (**d**) α_1_, α_2_ subunits in grey. (**e**) dimer-dimer interface (α_1_β_1_- α_1_β_1_) within the *A. marina* (α_1_β_1_)_3_ hexamer showing the position of the PCB chromophores (blue, PDB:1HA7). Differential residues at the dimer-dimer (**f**) and the β-β interface (**g**). The structures are coloured as follows: light pink = α_1_, light orange = α_2_, dark pink = β_1_, dark orange = β_2_

Finally, we sought to understand why α_1_β_1_ and α_2_β_2_ complexes form distinct entities and are unable to form mixed complexes. Sequence alignment of the two variants revealed 69% and 78% identity between the β and α subunits, respectively (Fig. 5a, b). For the β_1_ and β_2_ variants, differences were observed throughout the protein primary sequence with only helix E having large regions of conserved sequence (Fig. 5a). Structural models of the (α_1_β_1_)_3_ complex indicates that the sequence differences between the β_1_ and β_2_ variants largely map to the one face of the complex (Fig. 5c) with limited contacts with the α subunits. Conversely, between the α_1_ and α_2_ variants, sequence differences are focused within helix A, helix B, the end of helix E and the B-E loop region (Fig. 5d, e), regions which have been previously shown to be important for the interaction between individual αβ complexes within the (αβ)_3_ hexamer (Sound et al. 2026). Due to the known co-existence of all four isoforms within the crystal structure (5OOK)(Adir et al. 2001), AlphaFold predicted structures of the individual α_1_/α_2_ and β_1_/β_2_ subunits were used, and these structures aligned to the high resolution experimentally determined αβ complex from *A. platensis* (PDB:1HA7) and its corresponding modelled (αβ)_3_ PC complex. The αβ-αβ interaction interface within the (α_1_β_1_)_3_ hexamer is shown in Fig. 5e. One substitution that may primarily be responsible for the absence of mixed complex formation is Ser76 (β_1_) to His76 (β_2_) which interacts between the F and F’ helixes of the α chain (residues Ile112-Asn113 in α_1_, and Val112-Ser113 in α_2_) (Fig. 5f). Moreover, these residues are not too distant from the D-ring of the PCB chromophore that is known to change orientation within different PBP complexes to alter their absorbance properties (Fig. 5e). Another interface within the (αβ)_3_ complex is between the two neighbouring β subunits. The residues in the loops here are also remarkably different between the two isoforms; Asp17-Phe18 and Ile67-Gln68 in the β_2_ variant, and Ala17-Tyr18 and Cys67-Ala68 in the β_1_ variant (Fig. 5g).

## Conclusions

Here, we show the power of native MS to probe heterogeneity within PBS complexes. By monitoring the PBS core, we were able to detect low abundant components within APC complexes. This included the core linker, ApcC, which was detected attached to a single APC (αβ)_3_ complex, and the α_B_ subunit (ApcD) that was observed in up to three copies within a single APC (αβ)_3_ complex. The β_18_ subunit was also detected, however, in this instance only within an αβ APC dimer. The difference in stoichiometry for the αβ_18_ complex likely reflects its difference in stability in solution under the low ionic strength used for PBS complex disassembly, although complex dissociation during cell lysis may have been feasible.

Native MS revealed the PBPs, PC and APC, are unable to form mixed rings within the PBS structure. This is essential to ensure correct PBS assembly. We have previously shown that PC can form heterologous (αβ)_3_ containing αβ complexes from two distinct species (Sound et al. 2026). This is consistent with our native MS data here that shows the co-ability of α_1_β_1_ and α_2_β_2_ from *A. marina* to form complexes with PC from *A. platensis* (Fig. 4). This ability of the PBS ensures that it can evolve quickly to its surrounding environment. However, here, we show that when two PC variants are expressed within the genome of a single species, mixed PC complexes either cannot form (Fig. 4) or are below the detection limit of native MS. Intriguingly, upon alignment of α_1_β_1_ and α_2_β_2_ with the primary sequences of PCs from different species no general trend is apparent that can rationalise why mixed complexes occur with other species, yet within *A. marina* they do not (Fig. S8). Moreover, we envisage that global structural changes surrounding the αβ interface likely contribute to the co-positioning of each subunit with respect to one another rather than the influence of a single residue. Considering the presence of linkers in vivo, PBS rod assembly might be regulated differently. Regardless, our data shows that when two variants of PC are expressed within a single cyanobacterium, they function as distinct entities, creating a means to transfer energy in a unidirectional manner through the PBS towards photosystems I and II. Although further studies are needed, we envisage selective PBP complex formation to be a feature conserved throughout all types of PBS structures. Indeed, these findings will aid future studies that aim to elucidate when and to what extent heterogeneity within PBS is needed for effective light energy transfer.

## Supplementary Information

Below is the link to the electronic supplementary material.

Supplementary Material 1

## Data Availability

All native mass spectrometry data supporting this article is freely available via the University of Birmingham eData archive: 10.25500/edata.bham.00001516.
