# Supplementary material for: Heterogeneity within phycobilisomes is highly orchestrated
Source: Photosynth Res. 2026 Feb 9;164(1):13. doi: 10.1007/s11120-026-01202-8 (PMC12883523; doi:10.1007/s11120-026-01202-8)
Supplement: Supplementary file 1 — Supplementary Material 1 [file 11120_2026_1202_MOESM1_ESM.pdf]

# Heterogeneity within Phycobilisomes is Highly Orchestrated

Jaspreet K. Sound, Maayan Suissa Szlejf, Hannah E. Wedgwood, Noam Adir, Aneika C. Leney

Contents

**Fig. S1**: Purification of PBP from *A.platensis*…………………………………………………………..…. 1

**Fig. S2**: Purification of PBP from *A.marina* ……………………………………………………………………. 1

**Fig. S3**: Native mass spectra of PC and APC purified from *A. platensis* and *S. major*…………………………. 2

**Fig. S4**: Native mass spectra of αβ region of *A. marina* phycobiliprotein extract……………………..…...... 3

**Fig. S5**: Native mass spectra of $(\alpha\beta)_3$ region of *A. marina* phycobiliprotein extract……………………...…...... 3

**Fig. S6**: Native MS highlighting *A. marina* $\alpha_1\beta_1$ and $\alpha_2\beta_2$ PC isoforms form selective heterogenous complexes with PC from *A. platensis*……………………………………………………………………………………….3

**Fig. S5**: Native mass spectra of purified $\alpha_2\beta_2$ PC from *A. marina*, and $\alpha_2\beta_2$ PC mixed with αβ PC from *A. platensis* or αβ APC from *A. platensis*………………………………………………………………………....... 4

**Fig S6**: Alignment of PC sequences from different cyanobacterial strains……………………....................... 5

**Table S1**: Predicted vs observed molecular weights of PBP complexes…………………….......................... 6

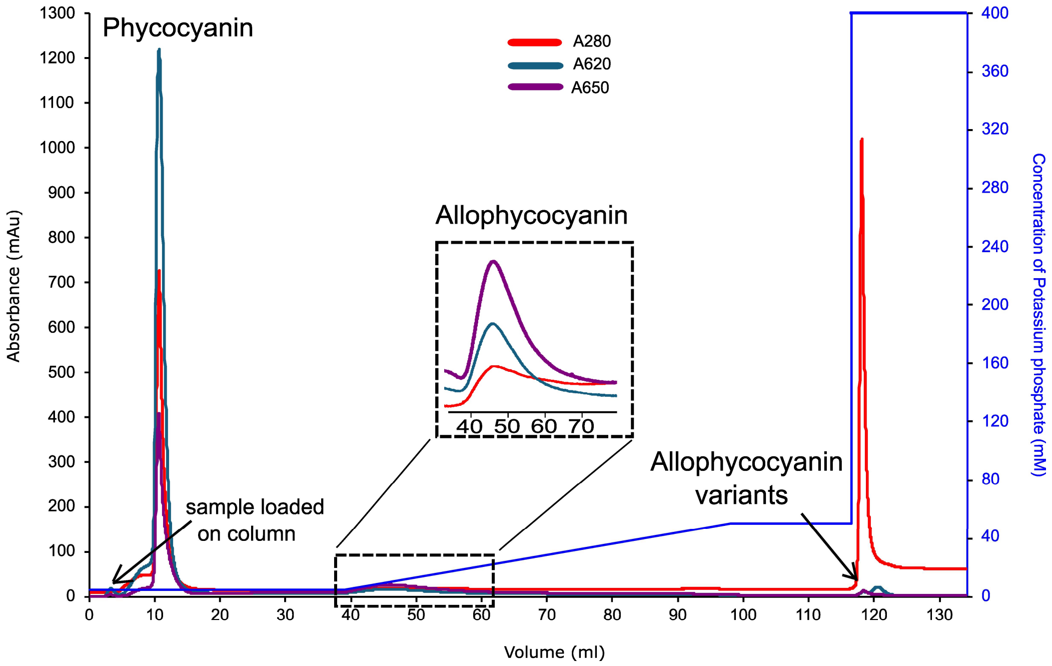


**Fig S1**: Example anion exchange chromatogram during purification of phycocyanin, allophycocyanin and allophycocyanin variants from *A. platensis* used for native MS analysis. The absorbance at 280 nm, 620 nm and 650 nm is shown in red, blue and purple, respectively, providing an indication of sample purity.

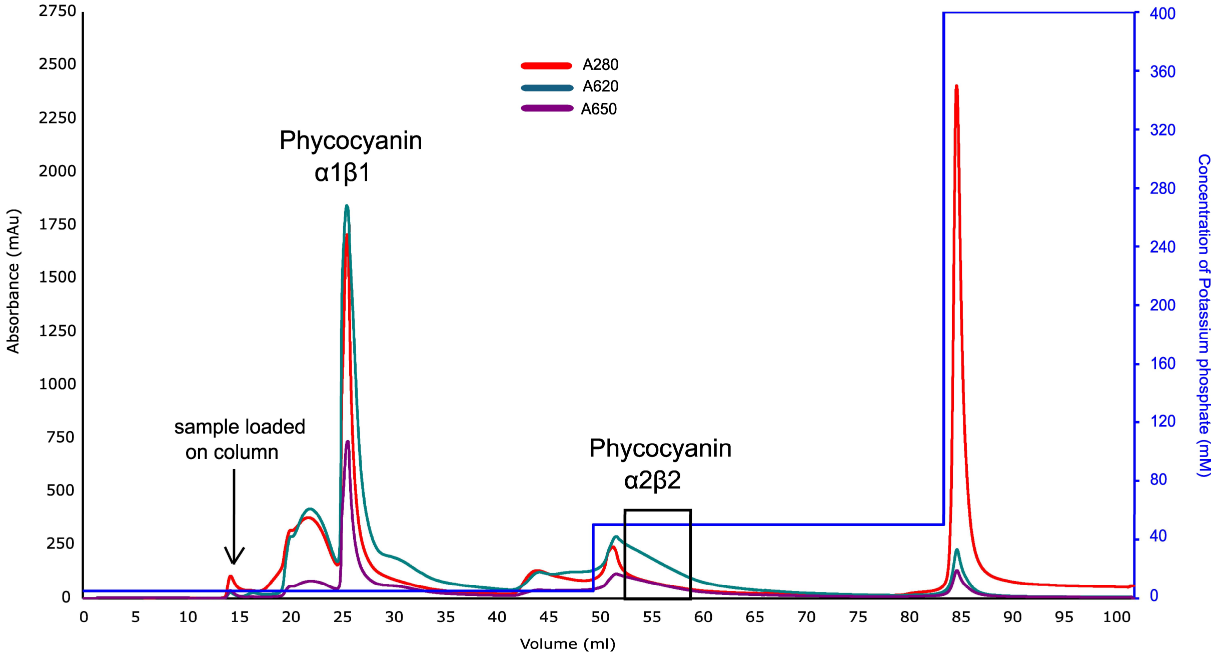


**Fig S2**: Example anion exchange chromatogram during purification of $\alpha_1\beta_1$ and $\alpha_2\beta_2$ phycocyanin from *A. marina*. The absorbance at 280 nm, 620 nm and 650 nm is shown in red, blue and purple, respectively. This provided an indication of sample purity that was further confirmed by native mass spectrometry analysis.

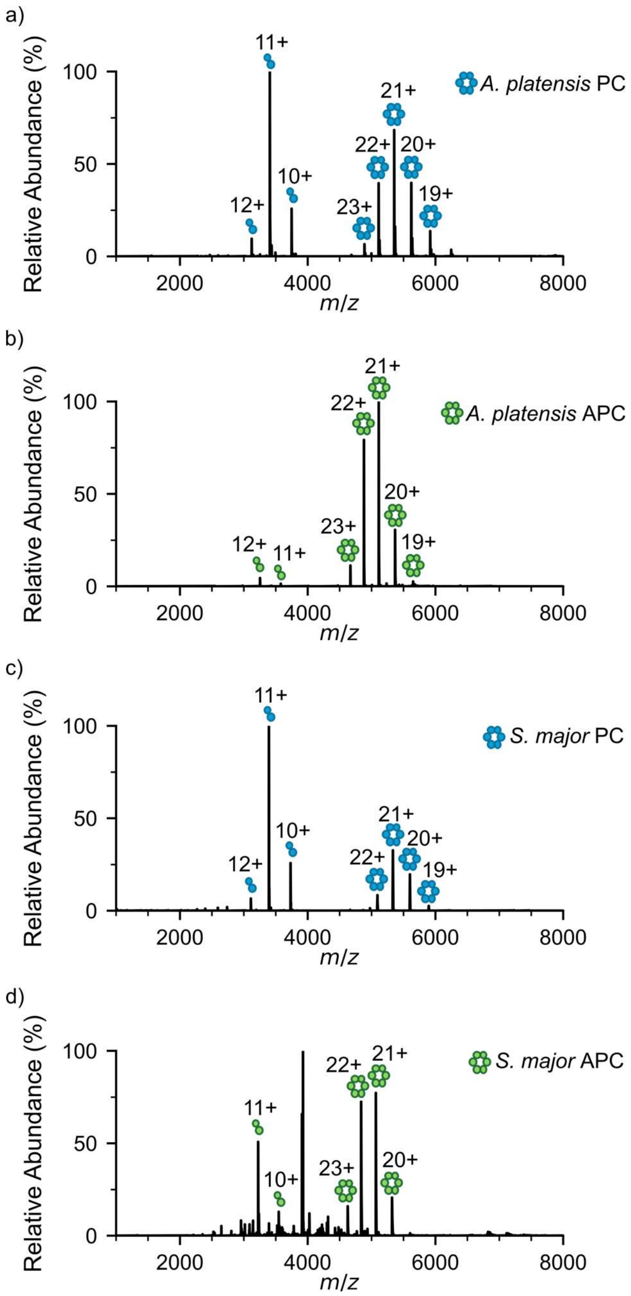


**Fig S**3: Native MS of PC (a,c) (blue) and APC (b,d) (green) purified from *A. platensis* (a, b) and *S. major* (c,d). Charge state distributions corresponding to the αβ and $(\alpha\beta)_3$ complexes are labelled. The peaks corresponding to PC and APC are the dominant peaks in the mass spectrum indicative of over > 75% complex purity in all cases.

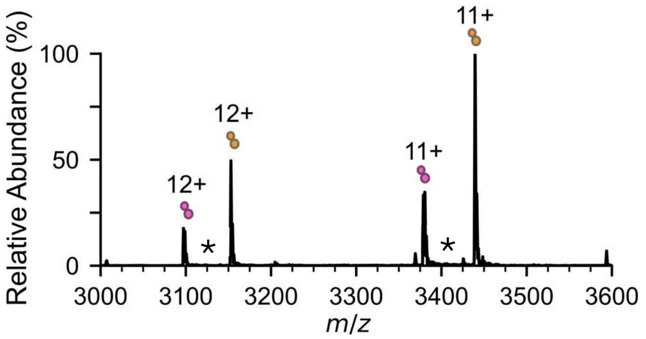


**Fig. S4**: Zoom in of the αβ region of the native MS of *A. marina* phycobiliprotein extract, highlighting the lack of APC and heterogeneous αβ complex formation between the $\alpha_1\beta_1$ (pink) and $\alpha_2\beta_2$ (yellow) PC isoforms. Theoretical m/z values where mixed αβ complexes would lie are indicated by a star.

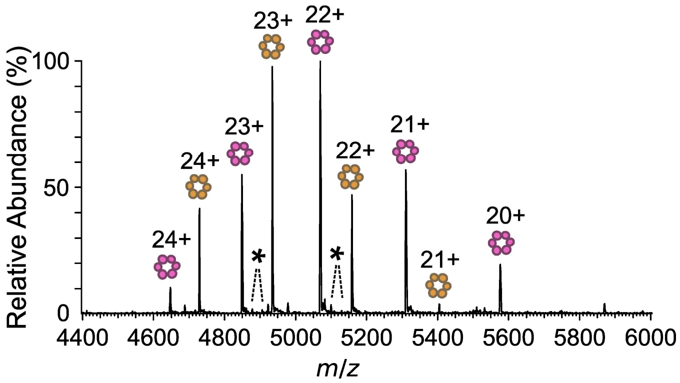


**Fig. S5**: Zoom in of the $(\alpha\beta)_3$ region of the native MS of *A. marina* phycobiliprotein extract, highlighting minimal mixed $(\alpha\beta)_3$ complex formation between the $\alpha_1\beta_1$ (pink) and $\alpha_2\beta_2$ (yellow) PC isoforms upon >48h of co-incubation. Theoretical m/z values where mixed αβ complexes would lie for the 22+ and 23+ charge states are indicated by a star.

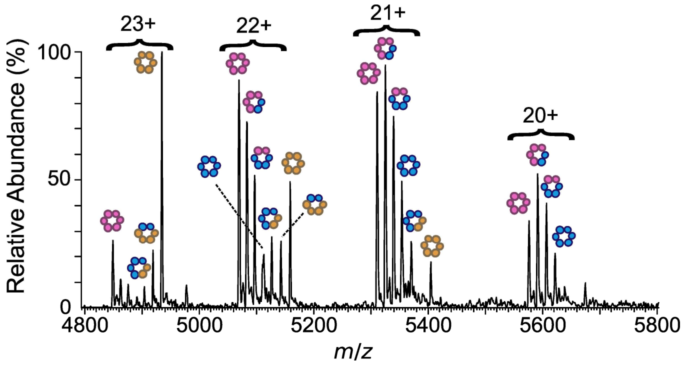


**Fig S6**: Native MS highlighting *A. marina* PC isoforms form selective heterogenous complexes. Fully annotated native MS of $(\alpha\beta)_3$ PC (blue) from *A. platensis* when mixed with $(\alpha_1\beta_1)_3$ (pink) and $(\alpha_2\beta_2)_3$ (orange) PC complexes from *A. marina*.

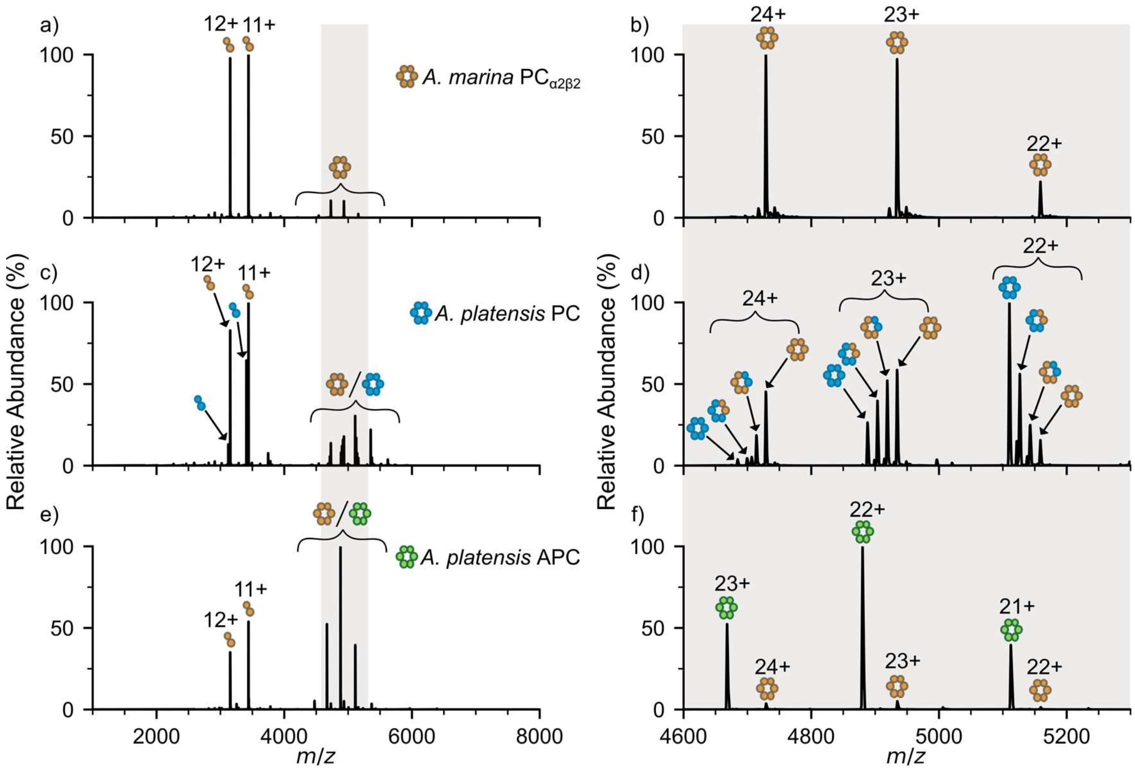


**Fig S7**: Native MS of purified $\alpha_2\beta_2$ PC from *A. marina* (a). Native MS of $\alpha_2\beta_2$ PC from *A. marina* mixed either with αβ PC from *A. platensis* (b) or αβ APC from *A. platensis*. Panels b, d and f are a zoom in of panels a, c and e, respectively. Note that mixed complexes formation is phycobiliprotein specific. Heterogeneous complexes form between $\alpha_2\beta_2$ PC and αβ PC (c,d) within < 1 h of incubation, but not between $\alpha_2\beta_2$ PC and αβ APC (e,f).

a) α subunit

```
                             10        20        30        40        50        60        70        80        90       100
                    ....|....|....|....|....|....|....|....|....|....|....|....|....|....|....|....|....|....|....|....|
A. marina α1      1 MQTPLIEAVSSADSQGRFLSSTELQVAFGRFRQAAASLDAAKTLNSKADSLADGAANAVYQKFPYTTQMTGSNYASTPEGKAKCVRDIGYYLRIISYCLI
A. marina α2      1 MKTPLTEALASADSQGKFLSSTELQVAFGRFRQASTGLQAARALSEKADSLASQAADAVYKKFPYTTSLQGNNYASTQRGKDKCVRDIGYYLRMITYCCV
A. platensis      1 MKTPLTEAVSIADSQGRFLSSTEIQVAFGRFRQAKAGLEAAKALTSKADSLISGAAQAVYNKFPYTTQMQGPNYAADQRGKDKCARDIGYYLRMVTYCLI
S. major          1 MKTPITEAISTADSQGRFLGNTELQAANGRFERAAASMEAARALTSKSTDLINGAANAVYQRFPYTTQMQGAQYAADQRGKDKCARDIGHYLRMVTYCLV
D. circinale      1 MKTPITEAIASADTQGRFLSNTELQAVNGRLVRAAASMEAARGLTANAQKLIDGATSAVYSKFPYTTSTQGNQFAADPRGKAKCARDVGHYLRIITYSLV
G. crepidinum     1 MKTPITEAIGAADTQGRFLSNTELQAINGRFDRAAASMEAARALTQKSQQLIDGAAQAVYQKFPYTTQMQGPQYAADSRGKSKCARDIGHYLRMVTYCLV
N. muscorum       1 MKTPLTEAVAAADSQGRFLSSTEIQIAFGRFRQAPASLEAAKSLTANAQRLTDGAAQAVYNKFPYTTQQQGPNYASTNTGKAKCARDIGYYLRIITYSLV
Kamptonema sp.    1 MKTPLTEAVTAADSQGRFLSSTELQVAFGRFRQATSGLEAAKSLSANAQRLTDGAAQAVYNKFPYTTQMQGNNYASDARGKAKCARDIGYYLRQITYCLV
P. priestleyi     1 MKTPITEAIAAADTQGRFLSNTELQAVNGRFDRAAASMEAARALTNNAQKLIDGAAQAVYSKFPYTTQMQGANFAATPEGKAKCSRDIGYYLRMVTYCLV
G. lithophora     1 MKTPLTEIIASADSEGRFLSNNELQSAFGRFGKAQAGLQAAKELTAKSDQLINQAAQAVYSKFPYTTQMQGNEYASDERGKAKCARDIGYYLRMVTYCLI
Synechococcus sp. 1 MKTPLTEAVAAADSQGRFLSNTELNAAFGRFERAKNALEAAKALTANADSLVNGAAQAVYNKFPYTTQMQGSNYASDARGKAKCARDIGYYLRMVTYCLV

                              110       120       130       140       150       160
                      ....|....|....|....|....|....|....|....|....|....|....|....|..
A. marina α1      101 AGGTGPLDDYLINGLAEINRTFDLSPSWYVEALKHIKANHGLSGDSAVEANSYIDYAIN-LS
A. marina α2      101 VGGTGPMDDYLVSGLAEINRTFDLSPSWYVEALKYIKANHGLSGDGAVEANSYIDYAINALS
A. platensis      101 AGGTGPMDEYLIAGIDEINRTFELSPSWYIEALKYIKANHGLSGDAAVEANSYLDYAINALS
S. major          101 AGGTGPMDEYLIAGLDEINRSFELSPSWYVEALKFIKASHGLSGQAANEANTYIDYAINALS
D. circinale      101 AGGTGPLDEFLIAGLAEVNAAFDLSPSWYVEALKSIKASHGLSGQAANEANTYIDYAINALS
G. crepidinum     101 SGGTGPMDEYLIAGLDEINRSFDLSPSWYVEALKHIKSNHGLQGQAANEANTYLDYAINALS
N. muscorum       101 VGGTGPLDDFLISGLAEINRTFDLSPSWYVEALKYIKANHGLSGDPAVEANSYIDYAINSLS
Kamptonema sp.    101 AGGTGPIDEYLIAGLDEINRTFELSPSWYVEALKYIKANHGLSGDAASEANSYIDYAINALS
P. priestleyi     101 AGGTGPMDEYLVAGLDEINRTFDLSPSWYVEALKNIKSSHGLSGQAANEANTYIDYAINALS
G. lithophora     101 VGGTGPMDEYLVAGLDEINSSFNLSPSWYVEALKSIKANHGLSGDSSVQANGFIDYAINALS
Synechococcus sp. 101 AGGTGPMDEYLVAGLDEINRTFELSPSWYVEALTYIKANHGVSGDAGVIANNYIDYAISALV
```

b) β subunit

```
                             10        20        30        40        50        60        70        80        90       100
                    ....|....|....|....|....|....|....|....|....|....|....|....|....|....|....|....|....|....|....|....|
A. marina β1      1 -MLDAFTKVVSQADTRGAYVSDAEVDALKAMVADANKRIDAVNRITGNASTIVANAARALFADQPQLCAPGGNAYTSRRMAACLRDMEIILRYVTYAVYT
A. marina β2      1 -MYDAFAKVVSQADARGDFLSDSQIAALQAVVSDGNKRMDVVNRITSNASTIVANAARDLFEEQPSLIQPGGNAYTHRRMAACLRDMEIILRYVTYAIFA
A. platensis      1 -MFDAFTKVVSQADTRGEMLSTAQIDALSQMVAESNKRLDAVNRITSNASTIVSNAARSLFAEQPQLIAPGGNAYTSRRMAACLRDMEIILRYVTYAVFA
S. major          1 -MFDAFTRVVSQADSRGEFLSTEQLDALAATVAAGSKRLDTVNRITSNASTIVTNAARALFAEQPQLISPGGNAYTNRRMAACLRDMEIILRYVTYATLA
D. circinale      1 MTLDVFSKVVSQADARGEFLSTEQLDALSAVVASGNKRLDAVNRITSNASAIVTNAARSLFEEQPQLIAPGGNAYTNRRNAACLRDMEIILRYVTYAAIA
G. crepidinum     1 -MLDAFAKVVSQADSRGEFLSNEQLDALTNMVKDGNKRLDTVNRITSNASSIVTDAARALFEEQPQLIQPGGNAYTNRRMAACLRDMEIILRYVTYAVMA
N. muscorum       1 MVLDAFAKVVSQADARGEYLSQAQLDALSAIVKDGNKRADTVNRITSNSSAIVANAARALFAEQPQLIAPGGNAYTSRRNAACLRDMEIILRYVTYAVFA
Kamptonema sp.    1 MSFDAFTKVVSQADARGEFLANSQLDALSSMVADGNKRIDAVNRITGNASSIVANAARSLFAEQPQLIAPGGNAYTHRRMAACLRDMEIILRYVTYATFA
P. priestleyi     1 -MLDAFAKVVSQADAKGEFLSNSQLDALSGMVKDGSKRLDTVNRITSNASTIVANAARTLFAEQPQLIAPGGNAYTNRRMAACLRDMEIILRYVTYATLA
G. lithophora     1 -MLDAFAKVVAQADTRGEFISTSQIDALSAMVAESNKRMDSVNRLTSNAAAIVTNAARSLFAEQPQLIQPGGNAYTNRRMAACLRDMEIILRYVTYAVLA
Synechococcus sp. 1 -MFDAFTKLVAQADARGEFLSPGQIDALGAMVAESNKRMDTVNRITSNASKIVTNAARDLFDQQPALIAPGGNAYTHRRMAACLRDMEIVLRYVTYAIFT

                              110       120       130       140       150       160       170
                      ....|....|....|....|....|....|....|....|....|....|....|....|....|....|...
A. marina α1      100 GDASVLNDRCLNGLRETYSALGVPGGSVAAGVQKMKEAAIEIANDPKGITQGDCSNLMAEIGSYFDLASSAVG
A. marina α2      100 GDASILEDRCLNGLKQTYQTLGVPTKSMSLSVSKMRDAALEIASDPNGVTQGDCSSLISEVSDYFDLAARAVG
A. platensis      100 GDASVLEDRCLNGLRETYLALGTPGSSVAVGVGKMKEAALAIVNDPAGITPGDCSALASEIASYFDRACAAVS
S. major          100 GDASVLEDRCLNGLRETYQALGVPGASVAAGVAKMKDAAVSAANDPNGITPGDCSQLMSEVAGYFDRAAAAVA
D. circinale      101 GDASVLDDRCLNGLRETYQALGTPGSSVAVGVGKMKEAAIAIVNDPNGITKGDCSSLVSELASYFDRAAAAVV
G. crepidinum     100 GDASVLDDRCLNGLRETYQALGVPGGSVAAGVQKMKDAAIKIANDPNGITQGDCSQLMSEVASYFDRAASAVA
N. muscorum       101 GDASVLNDRCLNGLRETYLALGTPGASVAVGVQKMKEAALAIAGDPNGITRGDCSALMAEVASYFDQAAAAVA
Kamptonema sp.    101 GDASILDDRCLNGLRETYQALGVPGGSVAAGVQKMKEAAIAIVNDTNGITRGDCSSLVSEIGGYFDRAASAVG
P. priestleyi     100 GDASVLDDRCLNGLRETYQALGVPGGSVAAGVQKMKEAAVGLANDPNGITKGDCSALMSEVSSYFDRAAAAVA
G. lithophora     100 GDASVLDDRCLNGLRETYQALGVPGASVAVGVQKMKEAAVSIVNDPTGITKGDCSQLVSEIASYFDRAAAAVV
Synechococcus sp. 100 GDASVLEDRCLNGLRETYLALGVPGASVAEGIRKMKDAAIAIANDRNGITPGDCSALMSEVGTYFDRAAAAVA
```

**Fig S8**: Alignment of PC a chain (a) and b chain (b) sequences from 10 selected cyanobacterial strains across the phylogenetic tree.

**Table S1.** Predicted versus observed molecular weights of phycobiliprotein complexes detected by native mass spectrometry. *denotes predicted molecular weight (MW) inclusive of predicted post-translational modifications.

| Protein Species | *Predicted MW (Da) | Observed MW (Da) | Charge States Detected |
|---|---|---|---|
| *A. platensis* | | | |
| PC ($\alpha\beta$) | 37,468 | 37,466 | 10+, 11+, 12+ |
| PC ($\alpha\beta$)$_3$ | 112,403 | 112,400 | 20+, 21+, 22+, 23+, 24+ |
| APC ($\alpha\beta$) | 35,778 | 35,777 | 11+, 12+ |
| APC ($\alpha\beta$)$_3$ | 107,333 | 107,339 | 20+, 21+, 22+, 23+, 24+ |
| APC ($\alpha\beta$)$_2$($\alpha$-B$\beta$)$_1$ | 107,944 | 107,945 | 23+, 24+, 25+ |
| APC ($\alpha\beta$)$_1$($\alpha$-B$\beta$)$_2$ | 108,553 | 108,559 | 23+, 24+, 25+ |
| APC ($\alpha$-B$\beta$)$_3$ | 109,163 | 109,167 | 24+, 25+ |
| APC ($\alpha\beta_{18}$) | 36,890 | 36,888 | 11+, 12+ |
| APC ($\alpha\beta$)$_3$ApcF | 115,109 | 115,119 | 22+, 23+, 24+ |
| *S. major* | | | |
| PC ($\alpha\beta$) | 37,307 | 37,307 | 10+, 11+, 12+ |
| PC ($\alpha\beta$)$_3$ | 111,921 | 111,925 | 18+, 19+, 20+, 21+, 22+, 23+, 24+, 25+ |
| APC ($\alpha\beta$)$_3$ | 106,375 | 106,377 | 20+, 21+, 22+, 23+ |
| *A. marina* | | | |
| PC$_1$ ($\alpha_1\beta_1$) | 37,171 | 37,173 | 11+, 12+ |
| PC$_1$ ($\alpha_1\beta_1$)$_3$ | 111,516 | 111,542 | 21+, 22+, 23+ |
| PC$_2$ ($\alpha_2\beta_2$) | 37,838 | 37,823 | 11+, 12+ |
| PC$_2$ ($\alpha_2\beta_2$)$_3$ | 113,513 | 113,469 | 21+, 22+, 23+, 24+ |
| *A. platensis + A. marina* | | | |
| PC ($\alpha\beta_{A.\ platensis}$)$_3$ | 112,403 | 112,406 | 20+, 21+, 22+, 23+ |
| PC ($\alpha\beta_{A.\ platensis}$)$_2$ ($\alpha_1\beta_{1\ A.\ marina}$)$_1$ | 112,107 | 112,118 | 20+, 21+, 22+, 23+ |
| PC ($\alpha\beta_{A.\ platensis}$)$_1$ ($\alpha_1\beta_{1\ A.\ marina}$)$_2$ | 111,811 | 111,829 | 21+, 22+, 23+ |
| PC ($\alpha_1\beta_{1\ A.\ marina}$)$_3$ | 111,516 | 111,542 | 21+, 22+, 23+ |
| PC ($\alpha\beta_{A.\ platensis}$)$_2$ ($\alpha_2\beta_{2\ A.\ marina}$)$_1$ | 112,773 | 112,764 | 21+, 22+, 23+, 24+ |
| PC ($\alpha\beta_{A.\ platensis}$)$_1$ ($\alpha_2\beta_{2\ A.\ marina}$)$_2$ | 113,143 | 113,140 | 21+, 22+, 23+, 24+ |
| PC ($\alpha_2\beta_{2\ A.\ marina}$)$_3$ | 113,513 | 113,469 | 21+, 22+, 23+, 24+ |
